# Draft Genome Sequence Analysis of the Genotype II African Swine Fever Virus from India

**DOI:** 10.1128/mra.00227-22

**Published:** 2022-10-26

**Authors:** Lukumoni Buragohain, Rupam Dutta, Arpita Bharali, Suparna Sen, Nagendra Nath Barman, Probodh Borah, Deep Prakash Saikia, Sachin Kumar, Yashpal Singh Malik, Satyam Pawar, Durlav Prasad Bora, Sophia M. Gogoi, Ashutosh Aasdev

**Affiliations:** a Department of Animal Biotechnology, College of Veterinary Science, Assam Agricultural University, Guwahati, India; b Department of Microbiology, College of Veterinary Science, Assam Agricultural University, Guwahati, India; c Department of Biosciences and Bioengineering, Indian Institute of Technology Guwahati, Guwahati, India; d College of Animal Biotechnology, Guru Angad Dev Veterinary and Animal Science, University (GADVASU), Ludhiana, India; e Department of Botany, Agricultural Trust’s Shardabaim Pawar Mahila Arts Commerce and Science College, Baramati, India; f CSIR-Center for Cellular & Molecular Biology, Hyderabad, India; KU Leuven

## Abstract

African swine fever virus (ASFV) entered the northeastern (NE) part of India early in 2020, causing huge economic loss to the piggery sector. Here, we are presenting a brief report on the draft genome sequence of an ASFV strain ABTCVSCK_ASF007 from Assam state of NE India belonging to genotype II.

## ANNOUNCEMENT

African swine fever (ASF) is a fatal contagious hemorrhagic viral disease of pigs with a mortality rate of up to 100%. The causative agent of ASF is the African swine fever virus (ASFV), which belongs to the genus *Asfivirus* and family *Asfarviridae* (1, 2). ASFV can infect both domestic and wild pigs irrespective of age. It is a multienveloped double-stranded DNA virus with icosahedral symmetry and has a large genome, which varies in length from 170 to 194 kbp (1) and encodes 150 to 167 open reading frames (2).

ASF is an alarming threat to the pig population across the world, especially in Asia where outbreaks are occurring continuously, including in the largest pig-producing country, China. In Asia, the first outbreak of ASF occurred in 2018 in China and subsequently spread over many other Asian countries, including Mongolia, Cambodia, Vietnam, and North Korea (3). In early 2020, the first devastating ASF outbreaks were reported from the northeastern states of India, particularly from Assam and Arunachal Pradesh. In April 2020, the outbreaks were confirmed as ASF by the Indian Council of Agricultural Research (ICAR)-National Institute of High-Security Animal Diseases, Bhopal, India (4).

On 22 July 2021, the ASF-suspected tissue samples (spleen, kidney, and liver) of a pig were collected from Village-Swetmada, Hazarikapara, under Sipajhar Development Block of Darrang District of Assam, and the sample was processed for diagnosis at the Advanced Animal Disease Diagnosis and Management Consortium (ADMaC) Laboratory, College of Veterinary Science (Guwahati, India). Genomic DNA was extracted from the pooled tissues using DNeasy blood and tissue kits (Qiagen, Germany). Initially, the presence of ASFV genomes in the DNA extract was confirmed by conventional PCR as per World Organization for Animal Health (OIE) guidelines (5). The ASFV-positive DNA extract was used for complete genome sequencing using next-generation sequencing by outsourcing (Eurofins, India). A paired-end sequencing library was prepared using a TruSeq Nano DNA library prep kit, as per the manufacturer’s instructions, and subjected to sequencing on an Illumina NextSeq500 platform using 2 × 150 bp chemistry. The raw sequenced data were processed by using Trimmomatic v 0.38 to obtain high-quality reads, which were further processed by aligning with the reference genome ASFV Belgium 2018/1 (6) (GenBank accession number LR536725.1) using BWA MEM v 0.717. A total 16,500,460 reads were generated, and the consensus sequence of the ASFV genome was generated with the help of Samtools (v0.1.18) mpileup utility. All the tools were run with default parameters.

**FIG 1:**
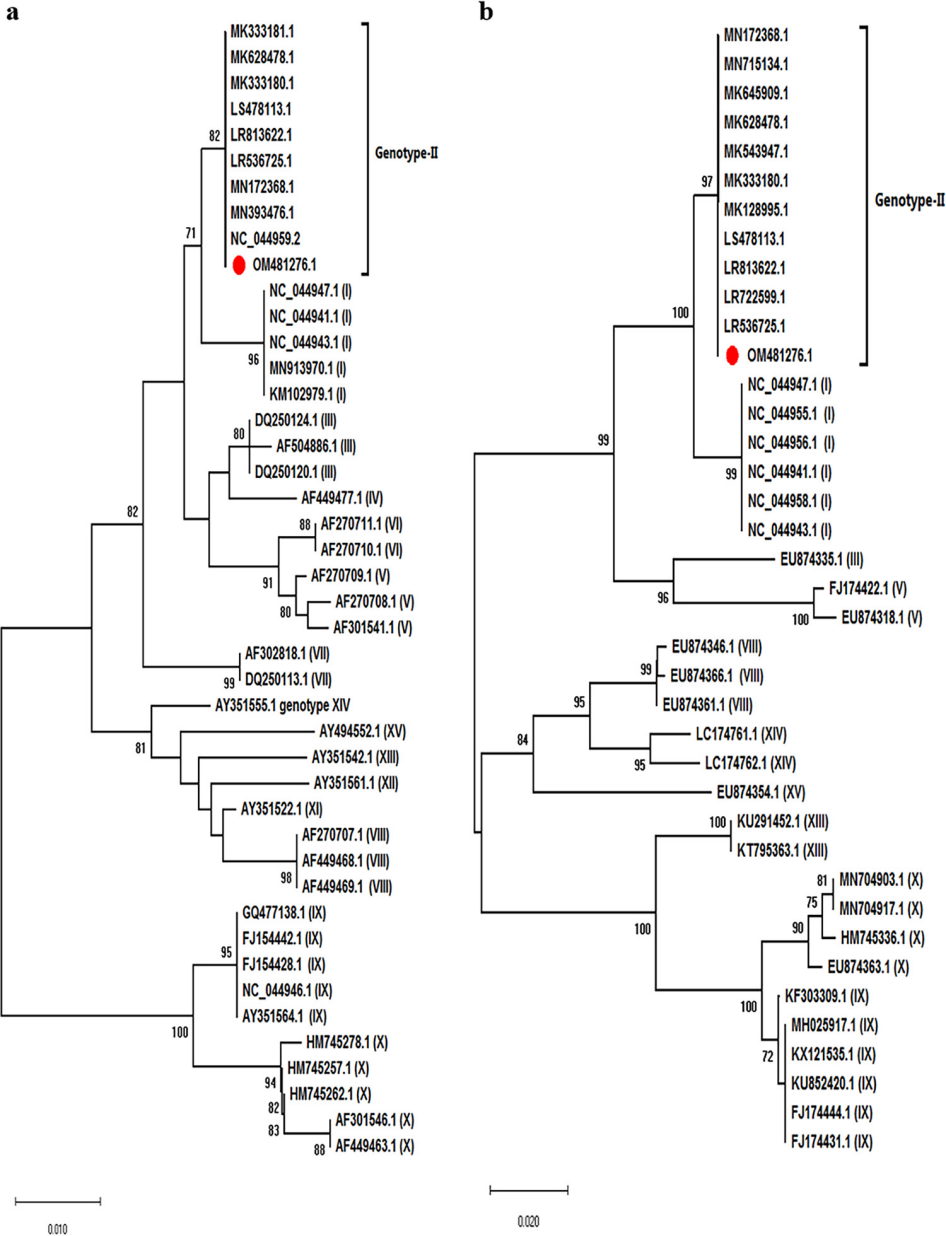
Phylogenetic analysis of ASFV based on *B646L* and *E183L* genes. Phylogenetic analysis was done in MEGA X software, and multiple sequence alignment of the nucleotide sequences was done with the CLUSTALW program. The phylogenetic trees were constructed by the neighbor-joining (NJ) method using a Kimura 2-parameter substitution model, and statistical significance was determined by 1,000 bootstraps. Gene sequences of different ASFV strains are represented by GenBank accession numbers with respective genotypes in parentheses. (a) A phylogenetic tree generated with a partial *B646L* gene and the ASFV strain ABTCVSCK_ASF007 (accession number OM481276.1, marked with a solid red circle) formed a clade with genotype II. (b) A phylogenetic tree constructed with the *E183L* gene also confirmed that the ASFV strain considered in this study belongs to genotype II of ASFV (highlighted with a solid red circle).

The final consensus ASFV genome sequence of the strain ABTCVSCK_ASF007 was found to be of 190,595 bp with GC content of 38.38% and 99.9% identity with its closest relative, ASFV Belgium 2018/1 (GenBank accession number LR536725.1). The putative number of open reading frames present in the ABTCVSCK_ASF007 strain was 195. Phylogenetic trees were constructed in MEGA X software based on the *B646L* (p72) and *E183L* (p54) genes, which revealed that the strain ABTCVSCK_ASF007 belongs to genotype II of ASFV (Fig. 1a and b). At the genome level, nucleotide identity of the ABTCVSCK_ASF007 strain was more than 99% with that of the ASFV isolates/strains belonging to genotype II that are available in the NCBI database, accessed on 8 February 2022.

African swine fever virus has recently entered India; thus, revealing the draft genome will strengthen the baseline data needed for epidemiological investigations and for the generation and design of diagnostics and vaccines in the near future.

### Data availability.

The near-complete genome sequence of African swine fever virus strain ABTCVSCK_ASF007 was deposited in GenBank under the accession number OM481276.1. The raw data were submitted to the SRA database under BioSample accession number PRJNA873208.

## References

[1] Alonso C, Borca M, Dixon L, Revilla Y, Rodriguez F, Escribano JM, ICTV Report Consortium. 2018. ICTV virus taxonomy profile: *Asfarviridae*. J Gen Virol 99:613–614. doi:10.1099/jgv.0.001049. 29565243 PMC12662184

[2] Dixon LK, Chapman DA, Netherton CL, Upton C. 2013. African swine fever virus replication and genomics. Virus Res 173:3–14. doi:10.1016/j.virusres.2012.10.020. 23142553

[3] Dixon L, Sun H, Roberts H. 2019. African swine fever. Antiviral Res 165:34–41. doi:10.1016/j.antiviral.2019.02.018. 30836106

[4] Rajukumar K, Senthilkumar D, Venkatesh G, Singh F, Patil VP, Kombiah S, Tosh C, Dubey CK, Sen A, Barman NN, Chakravarty A, Dutta B, Pegu SR, Bharali A, Singh VP. 2021. Genetic characterization of African swine fever virus from domestic pigs in India. Transbound Emerg Dis 68:2686–2692. doi:10.1111/tbed.13986. 33415828

[5] Agüero M, Fernández J, Romero L, Sánchez Mascaraque C, Arias M, Sánchez-Vizcaíno JM. 2003. Highly sensitive PCR assay for routine diagnosis of African swine fever virus in clinical samples. J Clin Microbiol 41:4431–4434. doi:10.1128/JCM.41.9.4431-4434.2003. 12958285 PMC193827

[6] Forth JH, Tignon M, Cay AB, Forth LF, Höper D, Blome S, Beer M. 2019. Comparative analysis of whole-genome sequence of African swine fever virus Belgium 2018/1. Emerg Infect Dis 25:1249–1252. doi:10.3201/eid2506.190286. 30907724 PMC6537744

